# Differences in classification of COPD group using COPD assessment test (CAT) or modified Medical Research Council (mMRC) dyspnea scores: a cross-sectional analyses

**DOI:** 10.1186/1471-2466-13-35

**Published:** 2013-06-03

**Authors:** Sunmin Kim, Jisun Oh, Yu-Il Kim, Hee-Jung Ban, Yong-Soo Kwon, In-Jae Oh, Kyu-Sik Kim, Young-Chul Kim, Sung-Chul Lim

**Affiliations:** 1 Division of Pulmonology, Department of Internal Medicine, Chonnam National University Hospital, 42 Jebong-ro, Donggu, Gwangju, 501-757, South Korea

**Keywords:** COPD, CAT, mMRC scores

## Abstract

**Background:**

The GOLD 2011 document proposed a new classification system for COPD combining symptom assessment by COPD assessment test (CAT) or modified Medical Research Council (mMRC) dyspnea scores, and exacerbation risk. We postulated that classification of COPD would be different by the symptom scale; CAT vs mMRC.

**Methods:**

Outpatients with COPD were enrolled from January to June in 2012. The patients were categorized into A, B, C, and D according to the GOLD 2011; patients were categorized twice with mMRC and CAT score for symptom assessment, respectively. Additionally, correlations between mMRC scores and each item of CAT scores were analyzed.

**Results:**

Classification of 257 patients using the CAT score vs mMRC scale was as follows. By using CAT score, 60 (23.3%) patients were assigned to group A, 55 (21.4%) to group B, 21 (8.2%) to group C, and 121 (47.1%) to group D. On the basis of the mMRC scale, 97 (37.7%) patients were assigned to group A, 18 (7.0%) to group B, 62 (24.1%) to group C, and 80 (31.1%) to group D. The kappa of agreement for the GOLD groups classified by CAT and mMRC was 0.510. The mMRC score displayed a wide range of correlation with each CAT item (r = 0.290 for sputum item to r = 0.731 for dyspnea item, p < 0.001).

**Conclusions:**

The classification of COPD produced by the mMRC or CAT score was not identical. Care should be taken when stratifying COPD patients with one symptom scale versus another according to the GOLD 2011 document.

## Background

Chronic obstructive pulmonary disease (COPD) is characterized by persistent airflow limitation [1]. The degree of airflow limitation is associated with many disease outcomes but was poorly predictive of dyspnea and quality of life [2-4]. Lung function alone does not explain the heterogeneous features of COPD [5,6]. Therefore, the Global Initiative for Chronic Obstructive Lung Disease (GOLD) 2011 document proposed a new classification system for COPD, combining symptom assessment and exacerbation risk including spirometry to identify disease severity [1].

For assessing symptoms, GOLD 2011 primarily recommends the use of the Modified.

British Medical Research Council (mMRC) questionnaire or the COPD Assessment Test (CAT). The mMRC scale is a 5-point (0–4) scale based on the severity of dyspnea [7]. The CAT comprises eight items relating to the severity of cough, sputum, dyspnea, chest tightness, capacity for exercise and activities, confidence, sleep quality and energy levels [8] while mMRC scale is a quantitative assessment tool only for breathlessness. These questionnaires are used to distinguish patients with less severe symptoms from patients with more severe symptoms; low vs high symptoms in the new GOLD 2011. However, a recent report indicated that group assignment of COPD patients could be different by the symptom scale that is used [9]; Han ML et al. used mMRC and SGRQ scores (as a surrogate of CAT scores) for assessing symptoms of COPD. The group assignment of COPD patients by mMRC vs SGRQ was not identical [9].

Hence, this study was performed to see whether the classification of COPD group according to GOLD 2011 would be identical irrespective of symptom scales (mMRC vs CAT score), and to evaluate the associations between two symptom scales.

## Methods

### Subjects

**Figure 1:**
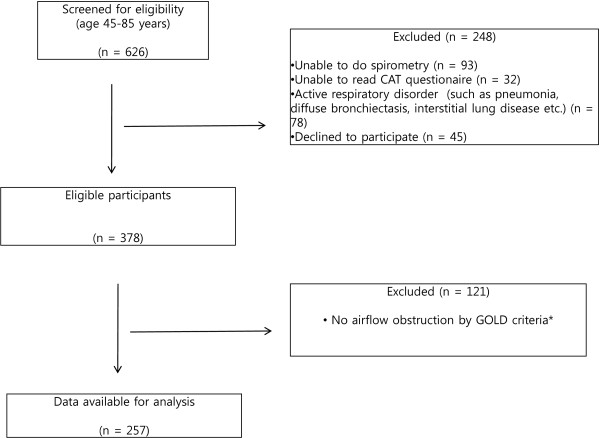
**Consort diagram of the study participants.** * Airflow obstruction was defined according to the GOLD criteria: a postbronchodilator FEV_1_/FVC (forced expiratory volume in 1s/forced vital capacity) ratio < 0.70.

Patients were recruited at outpatient clinics from the department of pulmonology, Chonnam National University hospital in South Korea. The flow chart for selecting patients is shown in Figure 1. All consecutive patients of the clinic were screened for eligibility from Jan to June, 2012 if they were aged 45–85 years. Patients must also have had the ability to undertake spirometry and answer to the questionnaire; CAT and mMRC. Those with active respiratory disorder such as pneumonia, diffuse bronchiectasis and interstitial lung disease were excluded. COPD was defined according to the GOLD criteria [1]: a postbronchodilator FEV_1_/FVC (forced expiratory volume in 1s/forced vital capacity) ratio below 0.70. Among 378 patients who responded the questionnaires and met the eligibility requirements, 121 subjects did not have airflow obstruction as defined by FEV1/FVC <0.70 and therefore they were excluded in this study. We analyzed the data from 257 patients with COPD. This study was approved by the ethics and review boards of Chonnam National University Hospital. All subjects gave informed consent to the work.

### Procedures

Symptoms were quantified with both the mMRC [7] and the CAT [8] (http://www.catestonline.org). The mMRC scale 0–4 was developed by the American Thoracic Society as a modification of the originally proposed British Medical Research Council dyspnea index (scale 1–5) [10]. The CAT consists of 8 items. Each item is scored from 0 to 5. An overall score is calculated by adding the score from each item; a total score ranging from 0 to 40. We determined the distribution of COPD patients with the mMRC and with the CAT. According to the GOLD 2011 classification [1], patients were stratified with either mMRC score (0–1 vs ≥2) or CAT score (<10 or ≥10) resulting in two low-symptom categories (A and C) and two high-symptom categories (B and D). Exacerbation risk was assessed with either FEV1% predicted (<50% or ≥50%), or COPD exacerbation history (0–1 vs ≥2) in the previous year to stratify patients into low-risk groups (A and B) versus high-risk groups(C and D). An exacerbation was defined as an acute event characterized by a worsening of the patient’s respiratory symptoms that is beyond normal day to day variations and leads to a change in medication [1,11]. The number of exacerbations in the previous year was taken from patients’ history at the same time when mMRC and CAT score were measured. Patients underwent standardized spirometry before and after the inhalation of 10 mg of salbutamol by nebulizer [12]. Finally, the patients with COPD were categorized into A, B, C, and D combining symptom assessment by COPD assessment test (CAT) or modified Medical Research Council (mMRC) dyspnea scores, and exacerbation risk according to the 2011 GOLD report.

### Statistical analyses

All statistical analyses were done with IBM SPSS Windows version 20.0. All data are presented as mean (SD) where appropriate. Kappa coefficient is used to interpret the extent of agreement between two symptom scales (mMRC vs CAT). The discussion of agreement in this study is based on kappa value as described previously in the literature in which k < 0.00 is “poor,” 0 < k < 0.02 is “slight,” 0.21 < k < 0.40 is “fair,” 0.41 < k < 0.60 is “moderate,” 0.61 < k < 0.80 is “substantial,” 0.81 < k < 1.00 is “almost perfect”, and k = 1 is “perfect” agreement [13]. The correlation between mMRC dyspnea score and CAT score was examined using the Spearman rank correlation coefficient (rho), because the MRC dyspnea score is an ordinal categorical variable. Differences were considered statistically significant when p < 0.05.

## Results

From January to June in 2012, 257 patients with COPD completed the questionnaire; CAT and mMRC dyspnea scale. Characteristics of subjects (mean age 67.4 ± 9.4 years; 203 men, 54 women) are summarized in Table 1. The mean body mass index (BMI) was 22.7 kg/m^2^. The majority of subjects were cigarette smokers (n = 182, 70.8%). Fifty subjects (19.5%) were high risks based on GOLD’s spirometric criteria (FEV1 < 50%), and 132 (51.4%) subjects were high risk according to the number of exacerbations of COPD (≥2 exacerbations in the previous year). Mean mMRC and CAT scores were 1.5 and 15.7, respectively.

**Table 1:** Clinical characteristics and demographics of patients (n = 257)

Characteristics*	**Mean or number of patients**	**SD or %**
Age (years)	67.4	9.4
Sex (M:F), n	203:54	79%:21%
BMI	22.7	4.1
Smoking status		
ever smoker, n	182	70.8%
never smoker, n	75	29.2%
FEV1, % predicted	74.6	24.8
FEV1 ≥ 50%, n	207	80.5%
FEV1 < 50%. n	50	19.5%
FEV1/FVC	0.56	0.12
Frequent exacerbator, n	132	51.4%
mMRC scores	1.5	1.1
CAT, total	15.7	9.3

*Values presented as mean (S.D.) unless otherwise indicated.

*Abbreviation: CAT* COPD Assessment Test, *mMRC* modified Medical Research Council, *BMI* Body Mass Index (kg/m^2^), *FEV1* Forced expiratory volume in 1 second, *FVC* Forced vital capacity.

Group assignments using the mMRC scale and CAT score were shown in Table 2 and 3. Among 257 COPD patients, using mMRC scale (Table 2), 97 (37.7%) patients were assigned to group A, 18 (7.0%) to group B, 62 (24.1%) to group C, and 80 (31.1%) to group D; on the basis of the CAT score (Table 3), 60 (23.3%) patients were assigned to group A, 55 (21.4%) to group B, 21 (8.2%) to group C, and 121 (47.1%) to group D. When the stratification of symptom group was done by CAT scores, the proportion of high symptom groups was increased. The kappa of agreement for the GOLD groups by CAT and mMRC was 0.510, suggesting moderate not substantial agreement. To investigate why symptoms groups were not identical between CAT and mMRC, we displayed the distributions of total CAT scores that were stratified into four groups at every mMRC level (Table 4). Within each mMRC level, there was a wide heterogeneous distribution of CAT scores. The mMRC score of 2 corresponded with a mean CAT score of 21 (SD 8), whereas the mMRC score of 1 corresponded to a mean CAT score of 13 (SD 6).

**Table 2:** Classifications of COPD groups using mMRC scores (n = 257)

High risk	62 (24.1%)	80 (31.1%)
Low risk	97 (37.7%)	18 (7.0%)
	mMRC 0-1	mMRC ≥ 2

**Table 3:** Classifications of COPD groups using CAT scores (n = 257)

High risk	21 (8.2%)	121 (47.1%)
Low risk	60(23.3%)	55(21.4%)
	CAT < 10	CAT ≥ 10

**Table 4:** Distributions of CAT total scores at each mMRC level (n = 257)

			**mMRC**				
			**0**	**1**	**2**	**3**	**4**
CAT total scores	0–9	Count	37	38	5	2	0
		% of Total	14.4%	14.8%	1.9%	.8%	0.0%
	10–19	Count	12	53	12	11	1
		% of Total	4.7%	20.6%	4.7%	4.3%	0.4%
	20–29	Count	2	18	14	23	7
		% of Total	0.8%	7.0%	5.4%	8.9%	2.7%
	30–40	Count	0	0	8	12	2
		% of Total	0.0%	0.0%	3.1%	4.7%	0.8%

The correlations between mMRC scores and each CAT item were shown in Table 5. The mMRC scores displayed a wide range of correlation with each CAT item (r = 0.290 ~ 0.731; p < 0.0001). Of the CAT items, the dyspnea item had the strongest correlation with the mMRC scores (r = 0.731, p < 0.001). However, the correlation between mMRC and the item of sputum in CAT was not strong (r = 0.290, p < 0.001).

**Table 5:** Correlations between mMRC and each item of CAT scores

		**mMRC**	
		**Spearman’s rho**	**P value**
Each item of CAT	Cough	.310^**^	<.0001
	sputum	.290^**^	<.0001
	Chest tightness	.345^**^	<.0001
	dyspnea	.731^**^	<.0001
	activities	.625^**^	<.0001
	confidence	.654^**^	<.0001
	Sleep quality	.381^**^	<.0001
	Energy level	.526^**^	<.0001

**Correlation is significant at the <0.01 level.

## Discussion

A main finding of this study was that the group classification of COPD produced by each symptom scale was not identical because the distributions of CAT scores were heterogeneous at every mMRC level. The group classification is important because treatment is recommended according to the groups of COPD in the 2011 GOLD.

The mMRC scale is widely used to measure breathlessness because of brevity and simplicity [7]. However, it is a unidimensional measurement to quantify only dyspnea. The CAT score is a multidimensional method, which assess 8 items; not only dyspnea but also other symptoms and health status [8,14]. It has proposed as a new tool for assessment of COPD health status in daily practice since it has the advantage of being easy to perform and it can be associated with clinically important variables (FEV1%, exacerbation as well as MRC dyspnea scale) [15,16]. And, the CAT has good repeatability [17] and is responsive to exacerbation onset and recovery [18].

GOLD 2011 recommends the CAT or mMRC scores to distinguish the symptom groups (high vs low symptoms). And, GOLD mentioned that it is unnecessary to use more than one symptom scale. However, a wide range of CAT scores was seen at each mMRC score in our study. The group assignment of COPD patients by each symptom scale was different. As the symptomatic cutpoint to differentiate the symptom groups, GOLD proposed either CAT score of 10 or mMRC score of 2. But, this study showed that an mMRC of 2 did not correspond with a CAT of 10. An mMRC of 1 was comparable to a CAT of 10. These results support a recent report [9] Han et al. used St George’s Respiratory Questionnaire (SGRQ) score as a surrogate for the CAT score to assess symptoms. The choice of symptom measure influenced category assignment of COPD. And, the mMRC score of 1 corresponded with an SGRQ of 25 that is similar with a CAT score of 10 [9].

The above findings suggest that refinement for differentiating symptom groups would be needed. The one refinement method is the change of cutpoint for mMRC from 2 to 1 because an mMRC of 1 corresponds to a CAT of 10. When the patients of our study were stratified using mMRC ≥1 as the symptomatic cutpoint, the kappa agreement between the groups by mMRC and CAT was more improved 0.510 into 0.649 (Table 6). However, the agreement between them was not perfect. This suggests that patients classified by the two symptom metrics will not be identical irrespective of changes of symptom’s cutpoint. This can be also supported by Han et al.’s report [9]. Another potential refinement method is as follows: To stratify symptom groups in COPD, both CAT and mMRC should be measured routinely. Then, symptom group would be determined by symptom scales showing the higher score like assessment of exacerbation risk using FEV1 and previous exacerbation history. This method can be considered in the future revision of COPD stratification. However, the different categories for both symptom and exacerbation assessment could be more demanding without treatment consequences. This issue also should be further studied in the future to improve the assessment and management of COPD.

**Table 6:** **Classifications of COPD groups using mMRC scores** (**cutpoint of mMRC** = **1**)* (**n** = **257**)

High risk	9 (3.5%)	133(51.8%)
Low risk	41 (16.0%)	74(28.8%)
	mMRC < 1	mMRC ≥ 1

*Kappa agreement with COPD groups classified by CAT scores was 0.649.

Secondary finding of this study is that all of each CAT item was found to have significant correlations with mMRC scores. However, it had a wide range of correlation coefficients. The mMRC score is the most strongly correlated with dyspnea item of CAT while the correlations of mMRC with other items of CAT were less strong. This is not a surprising result since mMRC is an indicator of breathlessness. Also, this could suggest that mMRC scale only for breathlessness cannot be identical with CAT score and it cannot be used as a surrogate of CAT score.

We acknowledge a major limitation of this study. The subjects were the patients who referred from primary clinics and were recruited from a single center. Our results, however, are comparable with a recent, multicenter study. An advantage of this study is that the mMRC and CAT were compared directly regarding to the recent GOLD report. The other weakness of this study is that the prospective analysis of exacerbation was not included. A prospective study will be needed to explore the clinical impacts of stratification of COPD on future exacerbations and treatments in patients with COPD.

## Conclusions

In conclusion, the GOLD 2011 recommends the use of mMRC or CAT scores for assessing symptoms. However, we showed that choice of symptom scale can alter group assignment of COPD because mMRC and CAT do not behave identically in distinguishing symptom groups. A refinement of the GOLD classification should be considered in the future.

## Abbreviations

COPD: Chronic obstructive pulmonary disease; CAT: COPD assessment test; mMRC scores: modified Medical Research Council (mMRC) dyspnea scores.

## Competing interests

The authors declare that they have no competing interests.

## Authors’ contributions

All of the authors participated in the conception and design of the study; the data collection; revising and approval of the manuscript. SK contributed to writing of the manuscript. YK was responsible for all phases of funding, study design, analysis and manuscript preparation. All authors have read and approved the final manuscript.

## Pre-publication history

The pre-publication history for this paper can be accessed here:

http://www.biomedcentral.com/1471-2466/13/35/prepub
